# Supplementary material for: UniPR1331: Small Eph/Ephrin Antagonist Beneficial in Intestinal Inflammation by Interfering with Type-B Signaling
Source: Pharmaceuticals (Basel). 2021 May 24;14(6):502. doi: 10.3390/ph14060502 (PMC8225182; doi:10.3390/ph14060502)

Figure S1: Effects of ephrin-A1-Fc on TNBS-induced inflammatory responses

Disease Activity Index (A), macroscopic score (B), colonic length (C), colonic thickness (D), colonic MPO (E) and lung MPO (F) activity assessed in vehicle-treated normal mice (S) and in TNBS-treated mice administered with vehicle (CNT), ephrin-A1-Fc 16 µg/kg (eph16) and 50 µg/kg (eph50) (n=5-15 independent values per group). *P<0.05 vs. S mice; #P<0.05 vs. CNT mice; one-way or two-way (DAI) ANOVA followed by Bonferroni's post-test; Kruskal-Wallis followed by Dunn's post-test (MS).

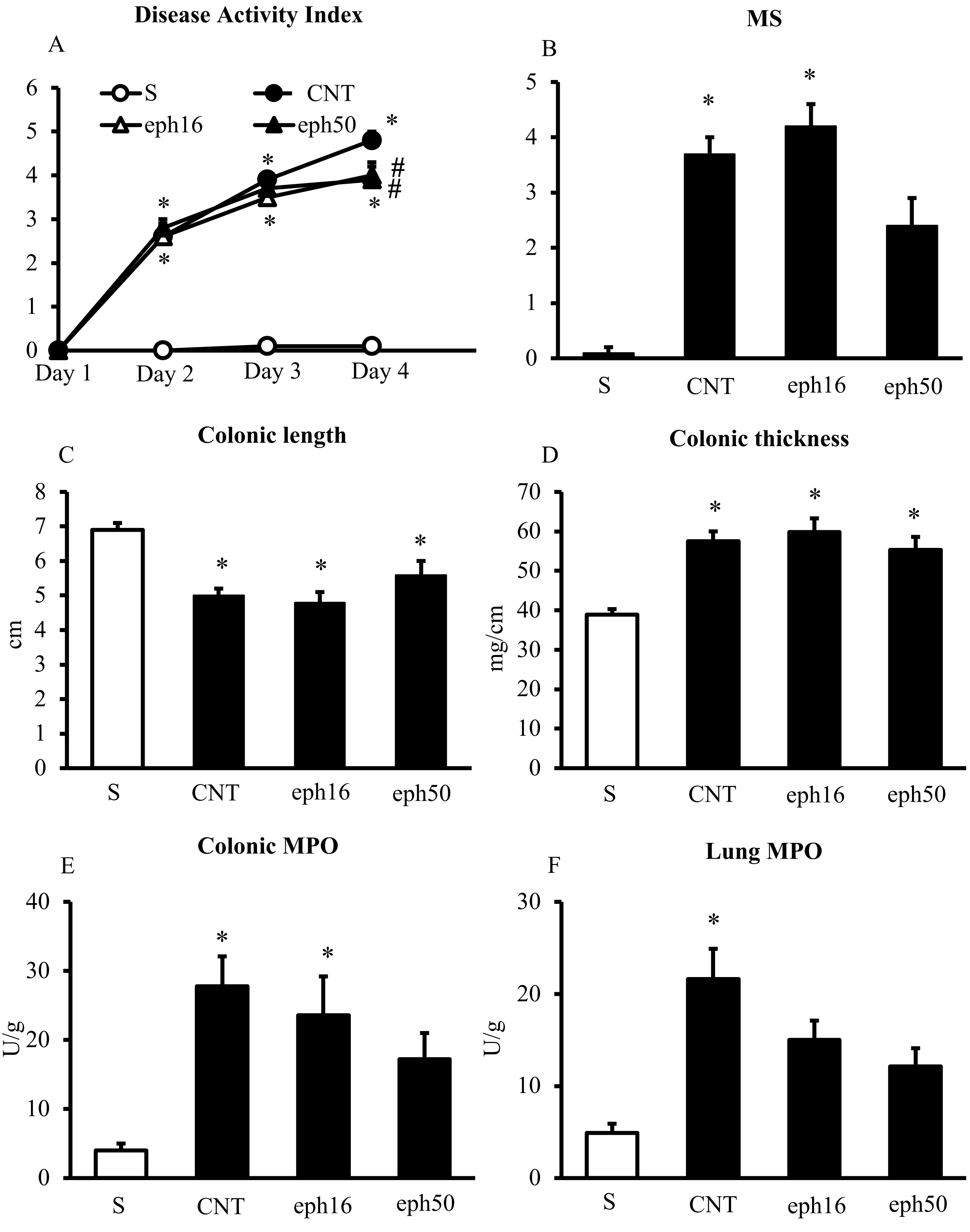

Supplement: Supplementary file 1 [file pharmaceuticals-14-00502-s001.zip › pharmaceuticals-1228672-supplementary.pdf]
